# Perinatal risk factors and 2-year neurodevelopmental outcome of early acute kidney injury in very preterm and very low birth weight infants

**DOI:** 10.1007/s00467-026-07170-4

**Published:** 2026-02-10

**Authors:** Isadora Beghetti, Ettore Benvenuti, Livia Lucchini, Silvia Martini, Annalisa Guarini, Alessandra Sansavini, Luigi Tommaso Corvaglia, Arianna Aceti

**Affiliations:** 1 Department of Medical and Surgical Sciences, University of Bologna, Via Massarenti 11, 40138 Bologna, Italy; 2 Neonatal Intensive Care Unit, IRCCS AOUBO, Bologna, Italy; 3 Department of Neurosciences, Faculty of Medicine and Psychology, Mental Health and Sensory Organs, Pediatric Unit, Sant’Andrea University Hospital, Sapienza University of Rome, Rome, Italy; 4 Department of Psychology “Renzo Canestrari”, University of Bologna, Bologna, Italy

**Keywords:** Acute kidney injury, Serum creatinine, Urine output, Very preterm-very low birth weight infants, Extremely low birth weight infants, Inotropes, Maternal hypertension

## Abstract

**Background:**

Acute kidney injury (AKI) is a significant complication for preterm infants, impacting both short- and long-term outcomes. This study aimed at identifying perinatal risk factors associated with early AKI and evaluating AKI’s impact on long-term outcomes.

**Methods:**

This retrospective cohort included 339 infants (born < 32 weeks gestational age or < 1500 g birth weight) admitted to a Level IV NICU between 2013 and 2017. AKI was defined either by serum creatinine (SCr) or urine output (UO) criteria. We examined gestational age, birth weight, perinatal factors, and medications (non-steroidal anti-inflammatory drugs [NSAIDs], inotropes) as predictors. Outcomes included early AKI, length of stay, growth at discharge, and neurodevelopment at 12 and 24 months corrected age. Univariate and multivariate logistic regression identified AKI risk factors, while linear regression assessed AKI’s impact on neurodevelopment.

**Results:**

AKI incidence varied by definition: AKI-SCr 42%, AKI-UO 7%. For AKI-SCr, extremely low birth weight (ELBW, OR 2.96, *p* = 0.002), NSAIDs (OR 2.14, *p* = 0.037), and inotropes (OR 2.26, *p* = 0.026) increased risk. Maternal hypertension (OR 0.51, *p* = 0.038) and female sex (OR 0.56, *p* = 0.037) were protective. For AKI-UO, ELBW (OR 6.52, *p* = 0.006) and inotropes (OR 3.60, *p* = 0.04) were the only risk factors. AKI-UO was linked to lower growth Z-scores and longer hospitalization. The relationship between AKI and poorer neurodevelopment disappeared after adjusting for neonatal comorbidities.

**Conclusions:**

Neonatal early AKI incidence and risk factors depend on diagnostic criteria. Low gestational age, birth weight, and drug exposure are key risk factors. Refining AKI definitions and conducting longitudinal outcome studies are essential.

**Graphical abstract:**

**Figure:**
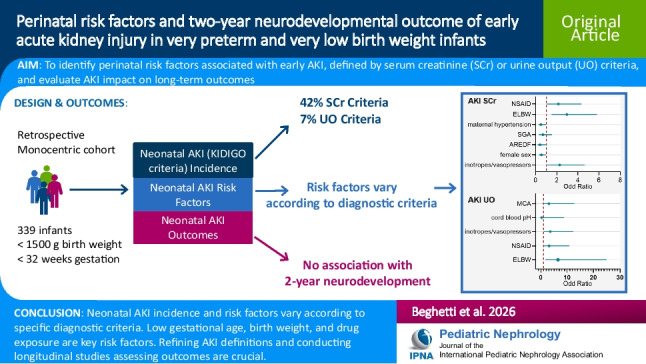
A higher resolution version of the Graphical abstract is available as Supplementary information

**Supplementary Information:**

The online version contains supplementary material available at 10.1007/s00467-026-07170-4.

## Introduction

Acute kidney injury (AKI) is recognized as a significant and frequently encountered complication in preterm infants, particularly those with very low birth weight (VLBW). The reported incidence of AKI in this vulnerable population during neonatal intensive care unit (NICU) hospitalization is substantial, ranging from 12 to 56% across various studies [1–3].

Neonatal AKI is associated with increased length of stay, higher mortality [1, 4–6], and increased hospital costs [7]. The consequences of AKI extend beyond the neonatal period: VLBW infants who experienced AKI in the NICU are more likely to have kidney dysfunction at 3–7 years of age compared to those without AKI [8], and severe AKI is associated with elevated blood pressure at 2 years of age [9]. Emerging evidence also suggests a potential link between AKI and poorer neurodevelopmental outcomes [10, 11]. The proposed mechanisms linking AKI to long-term morbidity are complex, involving shared hemodynamic vulnerability between the kidney and the developing brain and the systemic inflammatory cascade that drives postnatal growth restriction through protein catabolism and impaired anabolic signaling. Furthermore, severe AKI can lead to the accumulation of uremic toxins and labile electrolyte disturbances, which exert direct neurotoxic effects and compound the risk for cerebral injury. Therefore, understanding the independent impact of neonatal AKI on later neurodevelopmental and somatic outcomes is essential.

The modified Kidney Disease Improving Global Outcomes (KDIGO) criteria, encompassing both elevated serum creatinine (SCr) and reduced urine output (UO), are widely used to define AKI in neonates. To note, the diagnosis of AKI in VLBW infants presents unique challenges [1, 12]. Repeated blood sampling for SCr measurement can exacerbate anemia, a common problem in this population [13]. Additionally, defining the baseline SCr in the postnatal period is an iterative process with a focus on improvement, and accurate UO measurement, a key component of the oliguria criterion, is often technically difficult, leading to under documentation and potentially underdiagnosis of AKI [14]. In addition, AKI in newborns can be often non-oliguric, due to tubular immaturity, thus limiting the potential of UO monitoring in detecting anomalies in kidney function. Consequently, although several studies have investigated risk factors for AKI in very preterm infants, many have failed to incorporate UO into their AKI definition [3, 15, 16].

A comprehensive understanding of risk factors for AKI is crucial for improving neonatal care [17, 18]. Identifying these factors can minimize unnecessary blood sampling, optimize AKI detection, and facilitate the development of targeted interventions to reduce AKI risk and mitigate its long-term consequences. This, in turn, can enable personalized follow-up care for high-risk infants.

The primary aim of the study was to identify perinatal risk factors for early neonatal AKI, occurring in the first 10 days of life, defined using the two most common definitions available in the literature, which are based either on SCr or UO criteria. The secondary aim was to explore the impact of early neonatal AKI, irrespective of the specific definition applied, on long-term outcomes, including growth at hospital discharge and neurodevelopment assessed during the first two years of life.

## Methods

### Study design and ethics

A retrospective, single center, cohort study was conducted at a level IV NICU. The study was conducted in accordance with principles and standards of the Helsinki Declaration. The study protocol (study code: 76/2013/U/Sper/AOUBo) was approved by the local Institutional Review Board (CE AVEC, Bologna, Italy). Written informed consent was obtained from the infants’ parents or legal guardians for infants’ participation in the study and for data publication.

### Study population

Infants born very preterm (VLGA—gestational age [GA] < 32 weeks) and/or VLBW (birth weight [BW] < 1500 g) and admitted to the study NICU between January 2013 and December 2017 were included in the study. Infants with primary kidney disease or congenital malformations or genetic syndromes involving the kidney were excluded from the study.

### Definitions and classification of AKI

Changes in SCr and UO were used to diagnose neonatal AKI. The study focused on AKI occurring in the first 10 days of life (early AKI). Due to the retrospective nature of the study, the timing of SCr measurements analyzed for study purposes was determined by our institutional standard of care protocol that was in effect during the study period: routine SCr measurements were obtained on postnatal day 1, 2, 5, and again on day 7–10, with additional measurements collected as clinically indicated. Thus, all available SCr data collected during the first 10 days of life were included in the analysis. As per standard of care, UO was measured every 3 h via diaper weight. Neonatal AKI was diagnosed using the KDIGO criteria with neonatal modifications, and, for the study purposes, the two different definitions were used, either based on the SCr or UO criteria [1, 19].

According to SCr criteria, AKI was diagnosed when the absolute SCr value was > 0.5 mg/dl and then staged as follows: SCr ≥ 0.3 mg/dl above the baseline SCr or increased by 1.5–1.9 times compared to the previous lowest SCr value (Stage 1); SCr increased by 2.0–2.9 times above the baseline SCr (Stage 2); SCr increased ≥ 3 times above the baseline SCr or absolute SCr value ≥ 2.5 mg/dl (Stage 3). Comparison to the lowest prior SCr value is necessary because SCr values physiologically rise during the first 2 days of life and then decline over the first weeks after birth such that the “baseline” SCr is constantly changing. We defined the earliest baseline SCr as the value measured on day 2 of life [19].

According to UO criteria, AKI was diagnosed when the UO was ≤ 1 ml/kg/h and then staged as follows: UO > 0.5 and ≤ 1 mL/kg/hour (Stage1); > 0.3 and ≤ 0.5 mL/kg/hour (Stage 2), and ≤ 0.3 mL/kg/hour (Stage 3). Mean UO (ml/kg/hour) every 12-h period between day 2 and day 10 was calculated for study purposes. If an infant did not have at least 1 day with quantifiable UO in the medical record, it was considered to not have sufficient data to define AKI^UO.^

All AKI episodes that occurred during the first 10 days of life were considered.

AKI was diagnosed as AKI^SCr^ when defined by the abovementioned SCr criteria, or AKI^UO^ when defined by the UO criteria. Risk factors for AKI according to each definition were explored. Due to the nature of testing multiple AKI definitions, the definition of the control group shifted for each analysis. Specifically, for any given AKI definition being tested, the controls were defined as the infants who did not meet the criteria for that specific AKI definition. Infants meeting either AKI^SCr^ or AKI^UO^ criteria (one and/or the other) were classified as AKI^Any^, and this latter definition was used to assess the overall impact of AKI on long-term outcomes (growth and neurodevelopment).

### Data collection

#### Prenatal data and NICU stay

Data on maternal health, pregnancy outcomes, delivery characteristics, and neonatal health were collected from medical records. Specifically, the following prenatal and perinatal variables were recorded: prenatal Doppler ultrasound data (classified as absent or reverse end diastolic flow in the umbilical artery [AREDF], alterations in the blood flow in the ductus venosus [DV], alteration in the blood flow of the middle cerebral artery [MCA]), chorioamnionitis, maternal hypertension, antenatal steroid prophylaxis, administration of magnesium, fetal growth restriction (FGR), preterm prolonged rupture of membranes (pPROM), and type of delivery (vaginal delivery or cesarean section). Prenatal Doppler metrics reflect circulatory redistribution driven by chronic intrauterine hypoxia and placental insufficiency. This adaptive mechanism signals systemic stress and increased vulnerability to multi-organ injury, including cerebral, gastrointestinal, and neurodevelopmental complications [20]. At birth, the following neonatal variables were recorded: GA, sex, twin status, cord blood pH and base excess (BE), congenital malformations, and anthropometric measures at birth (BW, length, and head circumference [HC]), together with centiles and *Z*‐scores calculated using the Italian Neonatal Growth standards (INeS growth charts), which are recommended to assess auxological parameters at birth in the Italian neonatal population [21].

Major comorbidities occurring during hospitalization were also recorded and classified, using the definitions provided within the Vermont Oxford Network, which is an international registry collecting clinical data from very low birth weight, preterm infants worldwide [22]. Specifically, the following variables were collected: bronchopulmonary dysplasia (BPD) at any stage [23], respiratory distress syndrome (RDS) [24], respiratory support through mechanical ventilation (MV), intraventricular hemorrhage (IVH) at any stage [25], periventricular leukomalacia (PVL), patent ductus arteriosus (PDA), necrotizing enterocolitis (NEC) and its stage according to Bell’s classification [26], culture proven early and late onset sepsis (EOS/LOS), and retinopathy of prematurity (ROP) [27]. Exposure to potentially nephrotoxic drugs, such as non-steroidal anti-inflammatory drugs (NSAIDs), inotropes, and specific antibiotics (i.e., aminoglycosides and vancomycin), was also recorded. Auxological assessment at discharge was carried out using the INTERGROWTH‐21st charts, which are recommended for monitoring postnatal growth in preterm infants [28].

### Neurodevelopmental assessment

As per institutional clinical protocol (in line with the recommendations provided for preterm infants’ follow-up by the Italian Society of Neonatology**)**, all VLGA and/or VLBW infants admitted to the NICU who survived to discharge were scheduled to attend developmental follow-up at 3, 6, 12, 18, and 24 months corrected age (CA). Assessment of clinical conditions, growth, and neurodevelopment was performed at each visit [29].

Neurodevelopmental evaluation was performed at 12- and 24-months CA by a trained neuropsychologist using the revised Griffiths Mental Development Scale (GMDS-R) 0–2 years [30]. These scales investigate five developmental domains (locomotor, personal and social, hearing and language, eye and hand coordination, and performance), providing five sub-scale quotients and a general developmental quotient (GQ). The GQ was calculated using standardized score tables for the English infant population (mean ± SD, 100.5 ± 11.8), as no standardized data are available for the Italian population. Typical development was defined as a GQ score ≥ 88.7, mild neurodevelopmental impairment as a GQ score ranging from 88.6 to 76.9 (–1 to –2 SD below the normative mean), moderate neurodevelopmental impairment as a GQ score ranging from 76.8 to 65.1 (–2 to –3 SD below the normative mean), and severe neurodevelopmental impairment as a GQ score ≤ 65 (< –3 SD below the normative mean) [30, 31].

### Statistical analysis

All statistical analyses were carried out using IBM SPSS Statistics for Windows, Version 28.0 (IBM Corp.). Data distribution was examined through the Kolmogorov–Smirnov test; as most variables did not follow a normal distribution, non-parametric tests were used. Continuous variables are thus presented as median (interquartile range [IQR]) and dichotomous variables as number (percentage).

As for the primary outcome, prenatal and neonatal variables potentially associated with the development of AKI (according to each proposed definition) were first investigated through a univariate analysis. The Mann-Whitney *U* test was used for continuous variables, and the chi‐square test for dichotomous variables. Potential collinearity between independent variables to be included in the regression models was checked using the Pearson correlation coefficient or the point‐biserial correlation coefficient as appropriate. Correlation was defined as “strong” when correlation coefficients were above 0.6.

Variables which were significantly different between infants with and without AKI were included in one logistic regression model for each proposed AKI definition. For the secondary outcome, the relationship between AKI and neurodevelopment at 12 and 24 months was first explored using the Mann-Whitney *U* test for continuous variables and the chi-square test for dichotomous variables. To account for potential confounders, clinical variables known to impact neurodevelopment were subsequently included alongside AKI in two separate multivariable regression models (one for the 12-month and one for the 24-month assessment). A* p*-value < 0.05 was considered statistically significant.

## Results

Data from 339 infants were collected (Table 1); 30 infants lacked sufficient, reliable UO data between day 2 and day 10 to be accurately assessed by the UO AKI criteria and were consequently excluded from the UO-based AKI analysis. Three hundred and eighteen infants survived the neonatal period. Median GA was 30 weeks (IQR 27.7–31.4), and median BW was 1250 g (IQR 882.5 g); 31.1% of infants had an extremely low birth weight (ELBW, BW < 1000 g) and 24.1% had FGR. Females accounted for 46.5% of the population; most mothers (89.7%) had received antenatal steroid prophylaxis, while only a minority (36.7%) had received antenatal magnesium sulfate.

**Table 1:** Demographic and antenatal, perinatal, and postnatal clinical characteristics of the study population. Continuous variables are reported as median (IQR) and categorical variables as number [percentage]

Characteristics	*n* = 339
Gestational age (weeks)	30 (27.7; 31.4)
Mortality	21 [6.2%]
Inborn	295 [89.1%]
Antenatal steroids prophylaxis	262 [89.7%]
Magnesium sulfate prophylaxis	94 [36.7%]
Maternal hypertension	78 [26.7%]
Chorioamnionitis	5 [2.0%]
Birth weight (g)	1250 (882.5; 1440.5)
Birth weight *Z*-score	0 (− 1; 0.6)
Birth weight less 1000 g	103 [31.1%]
Fetal growth restriction	62 [24.1%]
SGA	65 [19.8%]
Length at birth	38 (35;40)
Length at birth *Z*-score	0 (− 1.2; 0.4)
Head circumference at birth	28 (25–29)
HC at birth *Z*-score	0 (− 1; 0.8)
Female sex	151 [46.5%]
Multiple birth	111 [33.5%]
Cesarean section	273 [82.5%]
PROM	84 [31.7%]
AREDF	57 [19.6%]
DV	11 [3.8%]
MCA	23 [7.9%]
Apgar score at 5 min	9 (1–10)
Cord blood pH	7.0 (7.23; 7.35)
BE	− 3 (− 5.73; − 0.85)
Major congenital anomalies	23 [6.8%]
NSAIDs	87 [27.27%]
Inotropes 10 days	87 [25.7%]
Early onset sepsis	21 [6.6%]
Late onset sepsis	57 [17.9%]
Necrotizing enterocolitis (Bell stage ≥ 2)	7 [2.2%]
Surgical necrotizing enterocolitis	6 [1.9%]
PDA	149 [46.3%]
Needing pharmacologic treatment	87 [27.3%]
Needing surgical treatment	14 [4.4%]
Retinopathy of prematurity	36 [11.6%]
Intraventricular hemorrhage	96 [29.9%]
Low grade (I–II)	81 [27.3%]
High grade (III–PVHI)	14 [4.7%]
Periventricular leukomalacia	10 [3.2%]
Respiratory distress syndrome	292 [90.1%]
Bronchopulmonary dysplasia	44 [14.1%]
Grade I	14 [34.1%]
Grade II	13 [31.7%]
Grade III	14 [34.1%]
Mechanical ventilation	120 [36.5%]
Duration of hospitalization (days)	42 (28; 63)
Post-menstrual age at discharge (days)	36 (35.3; 38.1)
Weight (g) at discharge	2020 (1820; 2345)
Discharge weight *Z*-score	− 1 (− 2.2; − 0.3)
Length at discharge	44 (42; 45)
Discharge length *Z*-score	− 2 (− 2.5; − 0.5)
Head Circumference at discharge	32 (30.6;33)
Discharge HC *Z*-score	− 1 (− 1.9; 0)

*SGA* small for gestational age, *HC* head circumference, *PROM* premature rupture of membranes, *AREDF* absent or reverse end-diastolic flow, *DV* ductus venosus, *MCA* middle cerebral artery, *BE* base excess, *PDA* patent ductus arteriosus, *PVHI* periventricular hemorrhagic infarction, *TEA* term equivalent age, *GQ* general quotient

Neonatal exposure to potentially nephrotoxic drugs was common. Consistent with the clinical protocol in place during the study period, all VLGA and VLBW infants received antibiotic prophylaxis including aminoglycosides (amikacin) during the first days of life. Furthermore, 27.3% of infants were administered NSAIDs (ibuprofen) for PDA pharmacological treatment according to local protocol. All VLGA and VLBW infants in this cohort received caffeine (same timing and same dosage for all included infants) as per clinical protocol and international guidelines [24]. Moreover, 25.7% of these infants necessitated inotropic/vasoactive support. The specific inotropic/vasoactive drugs and their prevalence were dobutamine (23.4%), dopamine (18.6%), milrinone (1.8%), epinephrine (1.2%), and norepinephrine (0.3%).

The incidence of AKI differed according to each proposed definition: AKI^SCr^ occurred in 141/339 (42%) infants and AKI^UO^ in 24/309 (7%) infants, of whom 7 had only AKI^UO^ and 17 had both AKI^UO^ and AKI^SCr^. As for disease severity, 75 infants (53%) had stage 1 AKI^SCr^, 51 (36%) had stage 2 AKI^SCr^, and 15 (11%) had stage 3 AKI^SCr^; in addition, 17 infants (71%) had stage 1 AKI^UO^ and 7 (29%) had stage 3 AKI^UO^.

### Risk factors for AKI

As shown in Table 2, low GA and BW (*p* < 0.001), ELBW (*p *< 0.001), and treatment with NSAIDs and inotropes (*p* < 0.001 for both) were all risk factors for both AKI^SCr^ and AKI^UO^. Additional risk factors differed according to AKI specific definition. Newborns with AKI^SCr^ were less frequently SGA, female, exposed to maternal hypertension, and were less likely to have AREDF in utero compared to controls (*p* < 0.05 for all comparisons). Conversely, newborns with AKI^UO^ had lower cord blood pH and BE compared to controls and had more frequently a history of antenatal Doppler alterations in the MCA compared to controls (*p* < 0.05).

**Table 2:** Characteristics for each specific AKI definition comparing AKI^SCr^ vs. No AKI^SCr^ and AKI^UO^ vs. No AKI^UO^

Characteristics	Participants, *n* (%)					
	AKI^SCr^ (*n* = 141)	No AKI^SCr^ (*n* = 198)	*p* value*	AKI^UO^ (*n* = 24)	No AKI^UO^ (*n* = 285)	*p* value*
Gestational age, median (IQR), weeks	28.71 (3.93)	30.71 (2.72)	** < *.001***	26.28 (4.57)	30.14 (3.43)	** < *.001***
Apgar score at 5 min, median (IQR)	8 (1)	9 (1)	** < *.001***	8 (4)	9 (1)	** < *.001***
Cord blood pH, median (IQR),	7.31 (.10)	7.28 (.11)	.11	7.25 (.21)	7.30 (.11)	***.02***
BE, median (IQR)	− 2.75 (5.26)	− 3.1(4.6)	.35	− 8.0 (8.4)	− 3.0 (4.6)	** < *.001***
Birth weight, median (IQR), g	1103.5 (580)	1335.0 (331)	** < *.001***	734.0 (288)	1298 (464)	** < *.001***
Birth weight less than 1000 g	62 (43.9)	31 (15.7)	** < *.001***	20 (83.3)	72 (25.3)	** < *.001***
Fetal growth restriction	23 (16.3)	38 (19.2)	.11	5 (20.8)	56 (19.6)	.78
SGA	19 (11.3)	44 (22.2)	***.02***	5 (20.8)	58 (20.4)	.79
AREDF	17 (12.0)	38 (19.2)	***.01***	6 (25)	49 (17.2)	.24
DV	7 (5.0)	3 (1.5)	.19	1 (4.2)	9 (3.2)	.52
MCA	8 (5.7)	14 (7.0)	.37	5 (20.8)	17 (6.0)	***.013***
Female sex	56 (39.7)	93 (46.9)	***.02***	9 (37.5)	137 (48.1)	.39
Multiple birth	43 (30.4)	61 (30.8)	.47	7 (29.2)	96 (34.4)	.82
Cesarean section	111 (78.7)	152 (76.8)	.07	18 (75)	242 (85)	.24
PROM	35 (24.8)	45 (22.7)	.59	4 (16.7)	75 (26.3)	.44
Antenatal steroids prophylaxis	119 (84.4)	134 (67.7)	.69	19 (79.2)	234 (82.1)	.48
Magnesium sulfate prophylaxis	47 (41.2)	45 (22.7)	.29	9 (37.5)	83 (29.1)	.31
Maternal hypertension	22 (15.6)	54 (27.3)	** < *.001***	6 (25)	70 (24.6)	.8
Chorioamnionitis	3 (2.6)	2 (1.0)	.66	0 (o)	5 (1.8)	1
NSAIDs	58 (50.8)	26 (13.1)	** < *.001***	18 (75)	68 (23.9)	** < *.001***
Inotropes 10 days	59 (51.8)	29 (14.6)	** < *.001***	16 (66.7)	67 (23.5)	** < *.001***

*BE* base excess, *SGA* small for gestational age, *AREDF* absent or reverse end-diastolic flow, *DV* ductus venosus, *MCA* middle cerebral artery, *PROM* premature rupture of membranes, *NSAIDs* non-steroidal anti-inflammatory drugs

*Based on Mann–Whitney *U* test for continuous variables, and chi‐square test for dichotomous variables

Variables associated with AKI in the univariate analysis were reviewed, and potential interactions among them were evaluated. Given the strong correlations observed between GA, BW, and ELBW (rho > 0.6, *p* = 0.01 for all pairwise comparisons), ELBW was selected for inclusion in all subsequent multiple logistic regression models as it captures the clinical subgroup with the highest vulnerability. Similarly, as cord blood pH and BE proved to be correlated (rho = 0.7, *p* = 0.01), only cord blood pH was included, reflecting the widespread clinical consensus that pH is the most consistent marker of immediate fetal compromise.

**Fig. 1:**
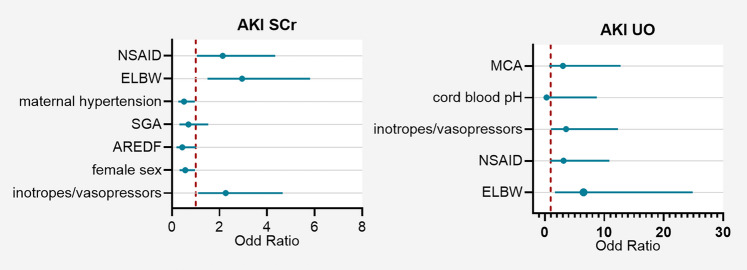
Multivariate analysis for risk factors associated with early acute kidney injury (AKI) according to serum creatinine criteria (AKI^SCr^) and to urinary output criteria (AKI^UO^)

In the final model (Fig. 1), only ELBW (OR 2.96, 95% CI 1.45–5.81, *p* = 0.002), NSAIDs (OR 2.14, 95% CI 1.05–4.35, *p* = 0.037), and inotropes/vasopressors administration (OR 2.26, 95% CI 1.10–4.66, *p* = 0.026) were found to be predictive of a higher risk of AKI^SCr^; conversely, maternal hypertension (OR 0.51, 95% CI 0.27–0.97, *p* = 0.038) and female sex (OR 0.56, 95% CI 0.32–0.98, *p* = 0.037) maintained their protective role against AKI^SCr^. As for AKI^UO^, ELBW (OR 6.52, 95% CI 1.71–24.87, *p* = 0.006) and inotropes/vasopressors exposure (OR 3.60, 95% CI 1.05–12.30, *p* = 0.04) were the only risk factors for neonatal AKI^UO^, after adjustment for other variables.

### Neonatal AKI and long-term outcomes

Both infants with AKI^SCr^ and AKI^UO^ experienced longer hospitalization compared to controls (median days 50 [IQR 36–85] vs. 36 [IQR 24–47] for AKI^SCr^ and 81 [44–116] vs. 41 [29–60] for AKI^UO^; *p* < 0.001 for both comparisons). At discharge, infants with AKI^UO^ had higher PMA (40 [37–42] vs. 36 [32–35] weeks, *p* = 0.004) and lower weight (−2 [−3.2/−1.2] vs. −1 [−2.2/−0.3], *p* = 0.04), length (−4 [−4.4/−1.7] vs. −1 [−2.4/−0.5], *p* = 0.002), and HC *Z*-scores (−2 [−3.2/−1.5] vs. −1 [−1.7/−0.05], *p* = 0.002) compared to non-AKI^UO^ infants. Growth *Z*-scores for infants with AKI^SCr^ at discharge were not significantly different compared to controls. Neurodevelopmental assessment was available for 224 infants at 12-months CA and for 207 infants at 24-months CA.

To exclude a potential bias related to missing neurodevelopmental data at 12- and 24-months CA assessments, baseline characteristics were compared between infants assessed at each of the two follow-up timepoints and those lost to follow-up. No difference in GA or in growth data, both at birth and discharge, nor in any of the main comorbidities of prematurity, was documented between groups at any of the two timepoints. At 12-months CA, median GQ (IQR) was 100 (91.5, 105.6); 19 (6%) infants had mild, 7 (2.2%) moderate, and 4 (1.3%) severe neurodevelopmental impairment. At 24-months CA, median GQ (IQR) was 95 (83, 103); 35 (11%) infants had mild, 19 (6%) moderate, and 14 (4.4%) severe neurodevelopmental impairment.

To assess the overall impact of AKI on neurodevelopment, a variable combining both types of AKI (AKI^Any^), defined according to AKI^SCr^ and/or AKI^UO^ criteria, was built. First, the relationship between AKI^Any^ and neurodevelopment was explored: a diagnosis of AKI, regardless of the specific definition used, was associated with lower GQ at both 12- and 24-months CA at the univariate analysis (*p* = 0.014 and *p* = 0.013, respectively).

To account for potential confounders, clinical variables which are known to impact neurodevelopment were tested for their potential relationship with neurodevelopmental scores in the present population, and those found to be significantly associated with neurodevelopment were included in two different linear logistic regression models (one for 12 months’ and the other for 24 months’ neurodevelopmental assessment) together with AKI.

Table 3 reports the results of the two linear regression models in which AKI and potential confounders were related to neurodevelopmental assessment at 12- and 24-months CA.

**Table 3:** Different models evaluating the effect of specific neonatal characteristics and acute kidney injury (AKI) on general quotient (GQ) at 12 months and 24 months corrected age. A *p* value < 0.05 was considered statistically significant

**Model 1: GQ at 12-months CA**						
Parameter	*B*	Standard error	Beta	B 95% confidence interval		*p*
				Min	Max	
(Constant)	103.866	1.157		101,585	106,147	< 0.001
AKI^Any^	− 1.908	1.413	− 0.083	− 4.692	0.876	0.178
IVH	− 4.015	1.538	− 0.159	− 7.046	− 0.985	**0.010**
PVL	− 16.789	4.395	− 0.232	− 25.451	− 8.128	** < 0.001**
LOS	− 0.379	2.118	− 0.012	− 4.553	3.795	0.858
BPD	− 6.612	2.396	− 0.190	− 11.333	− 1.890	**0.006**
ROP	0.374	2.232	0.010	− 4.024	4.772	0.867
PDA^pharm^	0.164	1.691	0.006	− 3.169	3.497	0.923
Weight *Z*-score at discharge	2.296	0.507	0.273	1.297	3.294	** < 0.001**
**Model 2: GQ at 24-months CA**						
Parameter	B	Standard error	Beta	B 95% confidence interval		*p*
				Min	Max	
(Constant)	97.933	1.573		94.832	101.034	< 0.001
AKI^Any^	− 2.675	2.027	− 0.086	− 6.672	1.321	0.188
PVL	− 30.778	6.298	− 0.302	− 43.177	− 18.380	** < 0.001**
LOS	− 7.620	2.938	− 0.179	− 13.412	− 1.827	**0.010**
BPD	− 2.991	3.101	− 0.067	− 9.104	3.122	0.336
PDA^pharm^	− 1.026	2.413	− 0.029	− 5.782	3.731	0.671
Weight *Z*-score at discharge	2.186	0.722	0.195	0.785	3.587	**0.002**

*GQ* general quotient, CA: corrected age, AKI^Any^: acute kidney injury episode meeting either serum creatinine or urinary output criteria, IVH: intraventricular hemorrhage, PVL: periventricular leukomalacia, LOS: late onset sepsis, BPD: bronchopulmonary dysplasia; ROP: retinopathy of prematurity, PDA^pharm^: patent ductus arteriosus needing pharmacologic treatment

After adjustment for confounders, AKI did not remain significantly associated with neurodevelopment, neither at 12- and 24-months CA. The variables which were found to be associated with GQ at 12-months CA were IVH, PVL, BPD, and a low *Z*-score for weight at discharge. The variables which remained significantly associated with GQ at 24-months CA at the regression analysis (Table 3) were PVL, LOS, and a low *Z*-score for weight at discharge.

## Discussion

This single-center retrospective cohort study investigated the incidence, risk factors, and long-term outcomes of early AKI in VLGA and/or VLBW infants, using both SCr and UO criteria. The study revealed a discrepancy in AKI incidence depending on the diagnostic criteria used, with 42% of infants experiencing AKI based on SCr criteria and only 7% based on UO criteria. The high incidence of AKI^SCr^ aligns with previous literature regarding AKI in preterm infants [3, 16]; conversely, the incidence of AKI^UO^ is lower compared to the few available studies reporting data according to this specific definition [2, 15]. The lower incidence of AKI^UO^, despite the implementation of standardized UO measurement protocols, suggests that oliguria may be less frequently observed, or more difficult to be accurately captured, in this population. Several factors could contribute to this discrepancy. Firstly, the technical difficulties associated with accurate UO measurement via diaper weighing [36], particularly in ELBW infants, may lead to the underestimation of the incidence of AKI^UO^ due to the overestimation of the true UO. Secondly, the intensive monitoring of UO may influence physician therapeutic decisions, potentially impacting kidney function and, consequently, UO itself. Furthermore, inconsistencies in UO evaluation across studies, such as variations in measurement intervals (e.g., 2-h vs. 3-h) and the lack of conclusive optimal UO cut-off values, contribute to the heterogeneity of findings [14].

In agreement with previous investigations, low GA and BW, particularly ELBW, were identified as significant risk factors for both AKI^SCr^ and AKI^UO^ [15, 16]. This association highlights the inherent vulnerability of immature kidney development and function in these infants [37]. Consistent with the existing literature, the administration of NSAIDs and inotropes emerged as independent risk factors for AKI^SCr^ [38]. Notably, inotropes/vasopressors exposure was also significantly associated with AKI^UO^. Several hypotheses may explain the observed relationship between inotropes/vasopressors exposure and AKI in our study. Firstly, certain inotropes may reduce kidney blood flow, particularly in the presence of inadequate circulating blood volume [32, 33]. Consequently, inotrope use could directly contribute to AKI. Secondly, inotrope administration may serve as a marker for hemodynamic instability, hypotension, and the severity of critical illness, potentially leading to AKI in this population. Irrespective of the specific mechanism, these findings underscore the necessity for meticulous kidney function monitoring in preterm infants exposed to these medications or experiencing these clinical conditions. Furthermore, we acknowledge that relying on a binary exposure variable for nephrotoxic drugs constitutes a methodological simplification. Future, prospectively designed studies are essential to systematically calculate and accurately assess the impact of the nephrotoxic burden on this vulnerable population.

Intriguingly, in line with previous reports, maternal hypertension and female infant sex were identified as protective factors for AKI^SCr^ [16, 34]. Reduced odds of neonatal AKI in infants exposed to maternal hypertension suggest a potential protective effect, either direct or mediated through an alternative pathway. Several hypotheses may explain this association. Firstly, maternal antihypertensive medication, and the subsequent control of maternal hypertension, could contribute to a reduction in neonatal AKI. Secondly, considering the heterogeneity of hypertensive disorders and the resulting fetal adaptation, it is possible that in subgroups of hypertensive pregnancies where placental function is not terminally compromised, the altered uteroplacental blood flow may enhance nutrient and oxygen delivery to the fetus, potentially promoting improved fetal kidney function or resilience [35]; furthermore, intermittent fetoplacental ischemia could precondition the neonatal kidneys to better tolerate ischemic events after birth [34]. Thirdly, it is plausible that these factors are not directly renoprotective, but rather reflect an association with meticulous management, including a complete course of antenatal corticosteroid prophylaxis and planned delivery, to mitigate risks for both mother and infant, and increased exposure to supportive care which may improve neonatal kidney hemodynamics. However, further investigation is warranted to elucidate the precise mechanisms underlying these findings.

The protective role of female sex is less clear and may reflect sex-specific differences in kidney physiology or response to injury [39]. Further research is warranted to explore this observation.

The study demonstrated that both AKI^SCr^ and AKI^UO^ were associated with prolonged hospitalization, highlighting the overall clinical impact of neonatal AKI. Moreover, AKI^UO^, but not AKI^SCr^, was associated with lower weight, length, and head circumference *Z*-scores at discharge. This suggests that AKI defined by UO criteria may reflect a more severe or prolonged insult to kidney function, potentially impacting growth and nutritional status.

Furthermore, in univariate analysis, any episode of AKI, irrespective of the diagnostic criteria employed, was associated with poorer neurodevelopmental outcomes at 12- and 24-months CA. However, in contrast to a recent study [10], after adjusting for pertinent neonatal comorbidities in a multivariate model, only PVL, LOS, and low weight *Z*-scores at discharge remained significantly associated with poorer neurodevelopment at 24-months CA. Discrepancies with previous studies may be attributed to differences in study population size and the incidence of AKI^UO^, which was higher in the cohort reported by Chen et al. [10]. Notably, De Mul et al. demonstrated that modified AKI definitions incorporating higher UO thresholds significantly improved the discriminative performance for predicting mortality [40]. Consequently, it could be argued that more accurate AKI definitions incorporating UO criteria and improved AKI detection methods may also enhance the discriminative performance for predicting outcomes beyond mortality. Regarding the lower incidence of AKI^UO^ observed in our cohort, this lower incidence may reflect the fact that AKI in newborns is often non-oliguric due to tubular immaturity, or that oliguria is a relatively late sign of kidney compromise in this population. Furthermore, our study utilized a 12-h UO measurement interval, as an adaptation of the standard KDIGO 24-h. This approach was chosen because the active, intensive monitoring of UO in our cohort may provide a more sensitive and real-time reflection of acute changes in kidney function and response to therapeutic interventions**.** Moreover, in our NICU, we actively monitor diuresis, and it is possible that, even though we chose a 12-h average period for study purposes, positive variation of UO may occur because of intercurrent medical managements that prevent prolonged oliguria. A very recent study assessing UO in VLBW infants in the first 28 days of life showed that UO is dynamic in the postnatal period and differs significantly between GA cohorts in the subgroup of extremely low gestational age neonates, suggesting the importance of an adaptation of UO thresholds and measurement protocols for neonatal AKI in preterm infants [14, 41]. Our use of the 12-h interval fits within the context of this need for optimized, dynamic detection methods that can best capture the rapid physiological shifts observed in the neonatal population.

A notable observation in our long-term follow-up was the apparent decline in median GQ score between the 12- and 24-month CA assessments. We hypothesize that this observation is most likely due to attrition bias (loss to follow-up). Specifically, infants with a favorable trajectory (higher scores) may have been selectively lost to follow-up, while those infants with more severe underlying neurodevelopmental issues (lower scores) remained actively engaged in the follow-up clinic for the 24-month assessment, leading to a lower median group score at the later time point due to selective retention. We reference that our baseline characteristics comparison did not show differences between infants who completed the 12-month versus the 24-month assessment, suggesting this may be a subtle effect of selective retention in follow-up.

It is important to acknowledge the limitations of this study. Our departmental protocol dictates routine SCr measurement on day of life 1, 2, 5, and 7–10. Additional SCr measurements were performed based on clinical indication. Critically ill infants, who are inherently at higher risk for AKI, likely have a greater number of measurements due to their clinical condition. This possible difference in testing frequency introduces a detection bias, potentially contributing to the high observed incidence of AKI^SCr^. The retrospective design does not allow us to establish causality and does not ensure that all potential variables which could act as confounders in the relationship between an early morbidity such as AKI and a long-term outcome as neurodevelopment, such as later AKI episodes, socioeconomic factors, family education, early support, and post-discharge growth, are recorded and considered. Furthermore, the statistical power of our analysis may be a constraint. The relatively low incidence of AKI^UO^ and the inclusion of numerous covariates in the multivariate models suggest that the study may have been underpowered to detect a significant association between AKI^UO^ and neurodevelopment. The single-center nature of the study may limit the generalizability of the findings. A fully time-dependent analysis of precise timing of AKI diagnosis and risk factors was not performed, and the duration of AKI episodes was not explicitly assessed, either of which could provide further insights into the relationship between AKI and outcomes [11, 16].

In conclusion, this study underscores the importance of utilizing both SCr and UO criteria in defining AKI in very preterm and/or VLBW infants, revealing differences in incidence based on the diagnostic approach. While low GA, BW, and exposure to nephrotoxic medications remain key risk factors, the role of inotropes/vasopressors, the protective association of maternal hypertension and female sex against AKI^SCr^ warrants further investigation. Notably, AKI^UO^ appears to correlate with more pronounced growth deficits and prolonged hospitalization, suggesting a potential marker for more severe kidney compromise. Although univariate analysis revealed an initial association between AKI and poorer neurodevelopmental outcomes, this relationship did not persist in the multivariate model, suggesting that other neonatal comorbidities exert a more significant influence on long-term neurodevelopment in this cohort. Future research should prioritize efforts to refine AKI definitions, improve risk stratification, and enhance AKI phenotyping for optimized detection in VLBW infants, while also exploring the underlying mechanisms of protective factors. Concurrently, longitudinal studies are crucial to elucidate the independent impact of AKI on later outcomes.

## Supplementary Information

Below is the link to the electronic supplementary material.

Graphical abstract (PPTX 138 KB)

## Data Availability

The datasets generated and/or analyzed during the current study will be made available by the corresponding author upon reasonable request, in accordance with local ethical committee policy at the time of study approval.
