# Supplementary material for: Perinatal risk factors and 2-year neurodevelopmental outcome of early acute kidney injury in very preterm and very low birth weight infants
Source: Pediatr Nephrol. 2026 Feb 10;41(8):2623–33. doi: 10.1007/s00467-026-07170-4 (PMC13337665; doi:10.1007/s00467-026-07170-4)
Supplement: Supplementary file 1 — Graphical abstract (PPTX 138 KB) [file 467_2026_7170_MOESM1_ESM.pptx]

## Slide 1
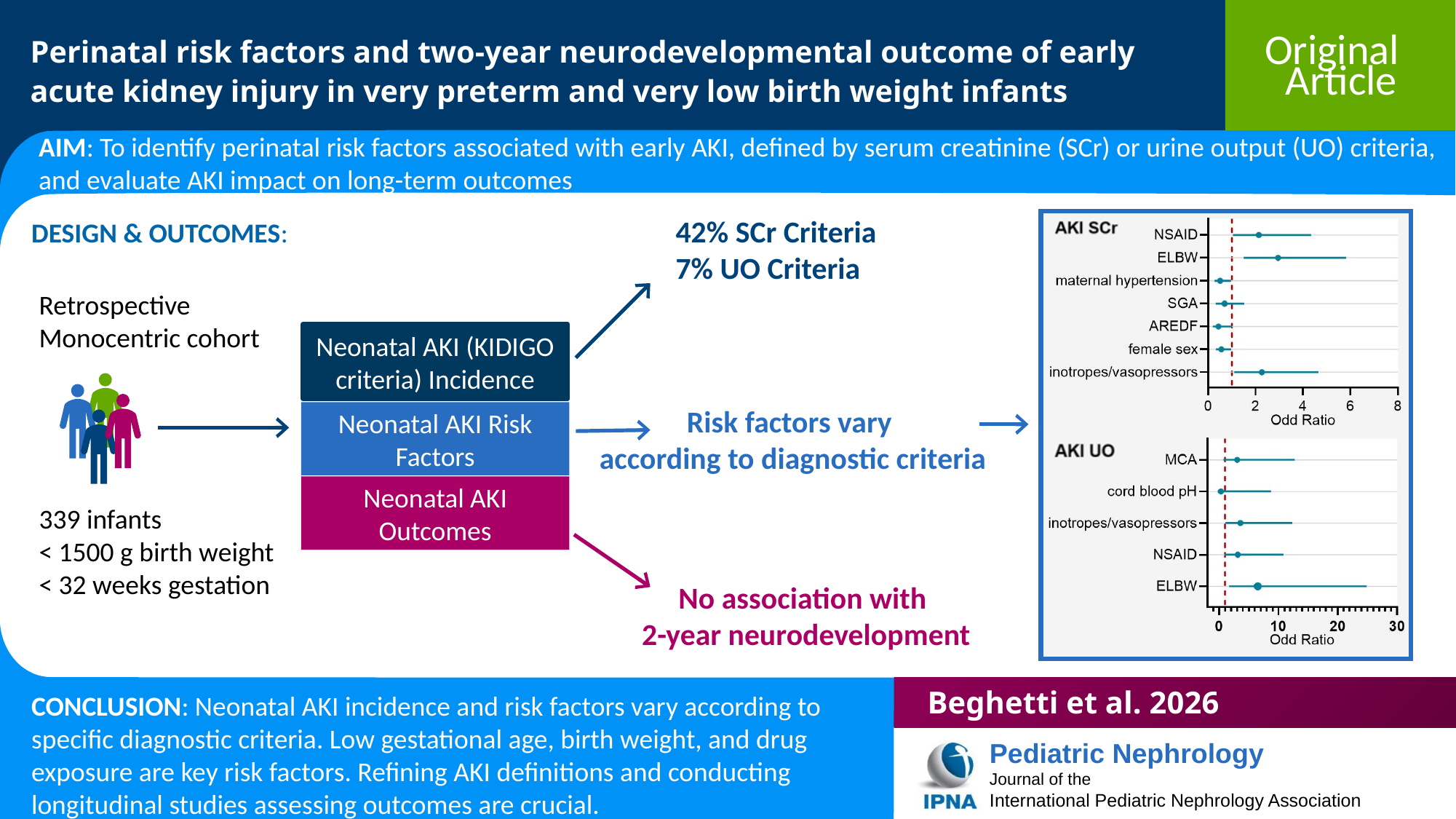

Perinatal risk factors and two-year neurodevelopmental outcome of early acute kidney injury in very preterm and very low birth weight infants
AIM: To identify perinatal risk factors associated with early AKI, defined by serum creatinine (SCr) or urine output (UO) criteria, and evaluate AKI impact on long-term outcomes
42% SCr Criteria
7% UO Criteria
DESIGN & OUTCOMES:
Retrospective
Monocentric cohort
Neonatal AKI (KIDIGO criteria) Incidence
Risk factors vary
according to diagnostic criteria
Neonatal AKI Risk Factors
Neonatal AKI Outcomes
339 infants
< 1500 g birth weight
< 32 weeks gestation
No association with
2-year neurodevelopment
Beghetti et al. 2026
CONCLUSION: Neonatal AKI incidence and risk factors vary according to specific diagnostic criteria. Low gestational age, birth weight, and drug exposure are key risk factors. Refining AKI definitions and conducting longitudinal studies assessing outcomes are crucial.
-----
